# Voice attractiveness affects cooperative behavior in the Stag Hunt Game: evidence from neural electrophysiology

**DOI:** 10.3389/fnins.2025.1576757

**Published:** 2025-03-28

**Authors:** Xianjia Wang, Wei Cui

**Affiliations:** ^1^Hubei Province Key Laboratory of Systems Science in Metallurgical Process, Wuhan University of Science and Technology, Wuhan, China; ^2^Economics and Management School, Wuhan University, Wuhan, China; ^3^College of Science, Wuhan University of Science and Technology, Wuhan, China

**Keywords:** decision making, ERO, ERP, Stag Hunt Game, voice attractiveness

## Abstract

**Introduction:**

The halo effect of attractiveness influences not only physical appearance but also vocal characteristics, impacting people’s lives and behaviors. Previous research has shown that voice attractiveness may affect decision-making and social interactions, but its influence on cooperative behavior has not been thoroughly investigated.

**Methods:**

This study used neurophysiological methods, specifically EEG, to examine the impact of voice attractiveness on cooperative behavior in the Stag Hunt Game. Participants played a two-person version of the game with a virtual partner whose voice was either highly attractive or less attractive. EEG data was recorded during the game, focusing on brain responses during the voice processing phase and outcome feedback phase.

**Results:**

The results revealed a “beauty premium” effect: participants were more likely to cooperate when paired with a highly attractive voice. Electrophysiological data showed that high-attractiveness voices elicited larger P2, P3, and LPC amplitudes and smaller theta oscillations during the voice processing phase. During the outcome feedback phase, both highly attractive voices and gain feedback resulted in smaller FRN amplitudes and larger P300 amplitudes. In contrast, under less attractive conditions, loss feedback triggered larger theta oscillations.

**Discussion:**

These findings suggest that voice attractiveness significantly influences cooperative behavior in social decision-making contexts. The neural responses indicate that the attractiveness of a voice can modulate both early sensory processing (P2, P3, LPC) and feedback evaluation (FRN, P300, theta). This study highlights the role of voice attractiveness in shaping cooperative behavior and provides new insights into the neurophysiological mechanisms underlying social decisions.

## 1 Introduction

Beautiful faces evoke positive emotions and trigger the desire to approach, thus creating attractiveness (Rhodes, 2006). People often believe that those with beautiful faces possess better personality traits, a stereotype known as the “what is beautiful is good” effect (Dion et al., 1972). Voices have also been shown to have the same effect, with highly attractive voices also being perceived as good (what sounds beautiful is good) (Zuckerman and Driver, 1989). This suggests that the halo effect of attractiveness is consistent across both visual and auditory channels, extensively influencing people’s daily lives.

Classical economics assumes a rational individual, where decisions made in economic activities are believed to solely pursue the maximization of one’s own benefit without considering other factors. However, in real life, individuals cannot achieve complete rationality, and their behavior is influenced by various factors. Attractiveness influences people’s various decision-making behaviors in social life, and individuals with highly attractive faces are typically more likely to find a partner, get a job, and win political elections (Bzdok et al., 2011; Langlois et al., 2000; Milazzo and Mattes, 2016; Poutvaara, 2014; Stockemer and Praino, 2017). Similarly, individuals with highly attractive voices also have certain advantages in social activities such as finding a partner, job seeking, and political elections (DeGroot and Kluemper, 2007; Hughes et al., 2004; Tigue et al., 2012).

Human society is a complex and dynamic system, and cooperative behavior helps maintain social order and promote the realization of common interests. The study of cooperative behavior not only reveals how humans make decisions in complex social situations but also helps us understand how different social signals influence cooperation. Individual attractiveness is an important social signal, and studying how attractiveness influences cooperation among individuals in a group is of great significance. In a labor market study by Hamermesh and Biddle (1993), it was shown that individuals with high attractiveness have certain advantages in the labor market, a phenomenon known as the “beauty premium.” Numerous studies suggest that the “beauty premium” phenomenon also exists in cooperative behavior. Mulford et al. (1998) designed a prisoner’s dilemma game, and the results indicated that people were more willing to cooperate with highly attractive partners. In a monetary decision-making task designed by Pandey and Zayas (2021), participants more frequently chose partners with high facial attractiveness, despite the risk of defection. In Solnick and Schweitzer (1999) Ultimatum Game experiment, partners with attractive appearances also gained higher rewards. Research on the impact of attractiveness on cooperative behavior has mostly focused on facial or physical attractiveness, with relatively little research on the effect of voice attractiveness. This paper will study the effect of voice attractiveness on cooperative behavior.

EEG technology has high temporal resolution, allowing for the exploration of neural mechanisms behind behavior on a precise time scale, typically using event-related potential (ERP) and event-related oscillation (ERO) techniques. Many studies using ERP techniques have revealed the neural processing of attractiveness in the brain. In a facial attractiveness judgment task, Schacht et al. (2008) found that both attractive and unattractive faces elicited larger early components around 150 ms and late positive components compared to moderately attractive faces. In an Ultimatum Game experiment by Ma et al. (2015), it was found that attractive faces induced larger LPC amplitudes compared to unattractive faces. In the study of voice attractiveness, Zhang et al. (2020) found that attractive voices elicited larger N1 amplitudes, smaller N2 amplitudes, and larger P3 amplitudes compared to unattractive voices. Studies using ERO techniques to investigate the neural mechanisms of attractiveness processing are relatively few, but some research suggests that the neural processing of facial beauty may involve slow-frequency oscillations, such as activity in the theta band (Zion-Golumbic et al., 2010). ERP technology can precisely capture the brain’s instantaneous response over a short period by recording time-locked potentials to specific stimuli (Fu et al., 2022). However, ERP technology cannot provide the frequency dimension of brain activity, and ERO technology compensates for this limitation by analyzing frequency oscillations in the brain to further explore the neural processes related to specific stimuli. By combining these two techniques, we can explore the brain’s neural responses to specific stimuli from both time and frequency dimensions. Therefore, this paper will combine ERP and ERO techniques to study the neural processing of voice attractiveness.

Outcome evaluation is an important stage in individual decision-making, where the brain encodes previous results to make better decisions (Platt, 2002). Many researchers have used ERP techniques to investigate the neural mechanisms of cooperative behavior in economic games, primarily analyzing two ERP components related to outcome evaluation: feedback-related negativity (FRN) and P300. The FRN component occurs in the early stages of outcome processing, with a negative peak approximately 200–350 ms after the presentation of the feedback stimulus (Holroyd and Coles, 2002; Proudfit, 2015). The FRN component serves as an indicator for distinguishing outcome valence, with negative feedback eliciting larger FRN amplitudes compared to positive feedback (Paul et al., 2025; Kobza et al., 2011; Yeung and Sanfey, 2004). The FRN component can also signal “expectation violation,” with studies showing that expectation violations lead to more negative FRN deflections (Gu et al., 2020; Pfabigan et al., 2011). After the early stage, P300 is another ERP component related to outcome evaluation, peaking approximately 300–600 ms after the outcome stimulus presentation (Zhang et al., 2022; Tao et al., 2023; Wu and Zhou, 2009). Some electrophysiological studies have shown that the P300 component is sensitive to outcome valence and reward magnitude, with positive outcomes or larger rewards eliciting larger P300 amplitudes (Li et al., 2025; Wu and Zhou, 2009; Yeung et al., 2005). Additionally, brain activity in the theta band (4-7 Hz) is associated with outcome evaluation, with negative feedback producing greater theta power than positive feedback (Cohen et al., 2007; Glazer et al., 2018; Luft, 2014).

**Figure 1:**
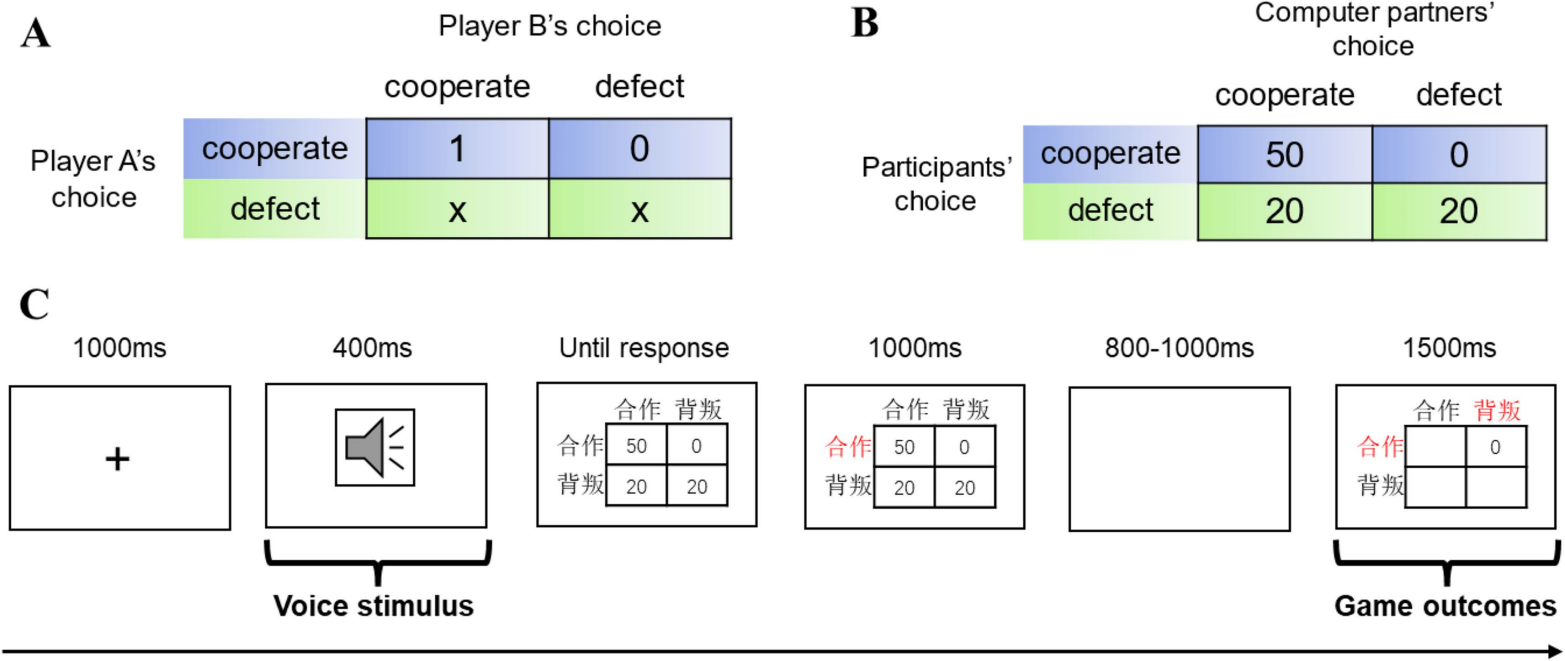
Payoff matrix of the two-person Stag Hunt Game and the experimental design of this study. **(A)** The payoff matrix of the general model of the two-person Stag Hunt Game. Rows represent player A’s choices, and columns represent player B’s choices. The numbers in the table represent the payoff that player A receives based on the different choices made by both players, where 0 < x < 1. **(B)** The payoff matrix of the two-person Stag Hunt Game in this experimental design. Rows represent the participant’s choices, and columns represent the computer partner’s choices. The numbers in the table represent the payoff (in yuan) that the participant receives based on different choices made by both parties. **(C)** Flowchart of a single trial. Participants first hear the voice of their game partner (high or low attractiveness), then make a choice between “cooperate(合作)” or “defect (背叛).” The choice made by the participant is highlighted in red. Finally, the results of both parties’ choices and the participant’s payoff for the trial are displayed. EEG data were recorded throughout the experiment.

The Stag Hunt Game is a classic model in game theory, often used in the study of cooperation problems (Skyrms, 2003; Yoshida et al., 2010; Deng and Zhang, 2022). This paper uses a two-person Stag Hunt Game model to explore the effect of voice attractiveness on cooperative behavior. The two-person Stag Hunt Game model involves two participants, where both must choose strategies under certain payoff rules, with each participant having two options: “cooperate” or “defect.” The payoff matrix for the two-person Stag Hunt Game is shown in Figure 1A, with the “rows” representing the choices of participant A and the “columns” representing the choices of participant B. The numbers in the table represent the payoff to participant A based on different choices, where 0 < x < 1. Choosing to “cooperate” is the risky “stag hunt” behavior, while choosing to “defect” is the risk-free “hare hunt” behavior. Only if both parties choose to “stag hunt” will the payoff be maximized, with both participants earning 1. However, if one participant chooses to “hare hunt,” the other participant’s payoff will be 0. The result of “stag hunt” is influenced by the partner’s behavior, but choosing to “hunt hare” is risk-free, and the outcome is not affected by the partner’s choice, yielding a steady payoff of x. The two-person Stag Hunt Game has two Nash equilibria: both choosing to cooperate represents the payoff-dominant equilibrium, which yields the highest payoff for both and is Pareto optimal. Both choosing to defect is the risk-dominant equilibrium, which avoids the risk of the other party not cooperating. The payoff matrix used in this study’s two-person Stag Hunt Game is shown in Figure 1B. When both the participant and their game partner choose to cooperate, the participant receives 50 yuan. If the participant chooses to cooperate while the game partner chooses to defect, the participant receives 0 yuan. If the participant chooses to defect, they will receive 20 yuan regardless of whether the game partner chooses to cooperate or defect.

In previous studies, researchers mostly used the prisoner’s dilemma game paradigm to explore the neural mechanisms of cooperative behavior. However, the prisoner’s dilemma game has only one Nash equilibrium, where both players choose to defect, making cooperation an unstable and unsustainable option that does not reflect the widespread phenomenon of mutual benefit and win-win situations in human society. The Stag Hunt Game is a typical cooperative game, where the greatest benefit can only be achieved if both players cooperate. Any defection by one party will reduce the rewards, while choosing cooperation is a stable and sustainable state, emphasizing the importance of mutual benefit and win-win situations. To the best of our knowledge, no study has explored the neural mechanisms of cooperative behavior in the Stag Hunt Game. Using this paradigm to investigate the neural mechanisms of voice attractiveness’ impact on cooperation can provide a fresh perspective in unraveling the mystery of human cooperation.

In summary, this paper aims to use the two-person Stag Hunt Game model as an example to comprehensively apply ERP and ERO techniques to explore the neural mechanisms of how voice attractiveness influences cooperative behavior. In this study, EEG data were collected from participants while they completed a two-person Stag Hunt Game task. Participants heard the voice of their game partner (high attractiveness or low attractiveness) before making a decision, and their behavior was recorded simultaneously. This study hypothesizes that under the influence of voices with different levels of attractiveness, the “beauty premium” effect will still exist, and participants will exhibit more cooperative behavior when faced with a highly attractive partner. In neurophysiology, the P2 component is thought to reflect “attentional capture” (Fernandes-Magalhaes et al., 2022), and has been widely reported in previous studies on salient stimuli such as attractive faces and emotional pictures (Liu et al., 2019; Schindler and Bublatzky, 2020). In an ERP study on facial attractiveness by Zhang and Deng (2012), it was found that attractive faces induced greater P2 amplitudes compared to unattractive faces. The P3 component is associated with attention allocation, and Zhang et al. (2020) found that attractive voices induced larger P3 amplitudes compared to unattractive voices. The LPC component is generally considered to reflect sustained attention allocation to motivationally relevant emotional stimuli. Some studies on facial attractiveness have found that attractive faces elicited larger LPC amplitudes compared to unattractive faces (Ma et al., 2015; Li et al., 2024). Therefore, during the voice stimulus presentation phase, we expect that high-attractiveness voices will induce larger P2 and P3 amplitudes compared to low-attractiveness voices. Theta oscillations are associated with conflict detection, and low-attractiveness voices may trigger psychological conflict in participants. Therefore, we expect that low-attractiveness voices will induce greater theta oscillations. Previous studies have shown that negative feedback induces larger FRN amplitudes (Paul et al., 2025; Kobza et al., 2011; Yeung and Sanfey, 2004) and theta oscillations (Cohen et al., 2007; Glazer et al., 2018; Luft, 2014), while positive outcomes induce larger P300 amplitudes (Li et al., 2025; Wu and Zhou, 2009; Yeung et al., 2005). We expect that during the outcome evaluation phase, loss feedback will induce larger FRN amplitudes, smaller P300 amplitudes, and higher theta band power compared to gain feedback.

## 2 Materials and methods

### 2.1 Participants

First, the sample size required for the experiment was estimated using G*Power software (version 3.1.9.7). This study used two statistical analysis methods: paired sample *t*-tests and 2 × 2 repeated measures ANOVA. For the paired sample t-test, the effect size dz. was set to 0.5, the statistical power was set to 0.8, and the significance level *α* was set to 0.05. The results indicated that at least 34 participants were required to achieve a medium effect size. For the 2 × 2 repeated measures ANOVA, the effect size f was set to 0.25, the statistical power was set to 0.8, and the significance level α was set to 0.05. The results indicated that 24 participants would be sufficient to achieve 80% statistical power with a medium effect size. A total of 34 undergraduate or graduate students from Wuhan University of Science and Technology (19 females) participated in this EEG experiment, aged between 19 and 28 years (*Mean* = 22.029 years, *SD* = 2.079 years). All participants were native Chinese speakers, right-handed, had normal or corrected-to-normal vision, no head trauma, and self-reported no mental illness or related family history. EEG data collection took place at the EEG lab of the School of Philosophy at Wuhan University. The experiment was approved by the Ethics Committee of Humanities and Social Sciences at Wuhan University, and all participants signed informed consent forms before the experiment.

### 2.2 Stimulus materials

In each trial of this experiment, participants were first presented with a voice stimulus. To allow participants to better perceive voice attractiveness and avoid influence from irrelevant variables such as tone and semantics, we used neutral vowels as the voice stimuli. The voice stimuli were selected from the “Geneva Faces and Voices Database” compiled by Ferdenzi et al. (2015), which contains audio recordings of 111 participants (61 females) producing three neutral vowel syllables (/i/, /a/, /o/). We excluded 4 audio files with noticeable noise and selected a total of 107 recordings (60 females). Using Praat software (version 6.4.10), each vowel was trimmed to 400 ms, and the sound intensity was uniformly adjusted to 70 dB, resulting in 321 voice audio files.

Before the EEG experiment began, we invited 32 undergraduate or graduate students (17 females, *Mean*_age_ = 23.250 years, *SD*_age_ = 2.437 years) from Wuhan University of Science and Technology, who did not participate in the EEG experiment, to rate the attractiveness of the 321 voice samples. The rating scale had 7 levels, with 1 representing the lowest attractiveness and 7 representing the highest. Attractiveness increased progressively from 1 to 7, and each participant received 10 yuan as compensation. Finally, we selected the 40 voice samples with the highest attractiveness scores (20 females) and the 40 voice samples with the lowest attractiveness scores (20 females) as the voice stimuli for the EEG experiment. The attractiveness ratings of the two groups of voice samples were compared using a paired sample t-test, which revealed a significant difference in attractiveness ratings [*Mean*_high attractiveness_ = 4.455, *Mean*_low attractiveness_ = 3.246, *t*(31) = 10.836, *p* < 0.001].

### 2.3 Experimental procedure

Each participant was placed in a soundproof, temperature-controlled, and electromagnetically shielded room to conduct the EEG experiment. The participant sat comfortably in a chair with a 22-inch LCD monitor in front of them, approximately 100 cm away. The experimental tasks were displayed on Figure 1C, and a keyboard was provided for making choices, with speakers presenting the voice stimuli. Participants were instructed to read the task description, which detailed the rules of the two-person Stag Hunt Game and the reward calculation method. Then, preparations were made, including fitting the EEG cap and injecting conductive gel into the electrodes. The experimental task was programmed using Eprime software (version 2.0). To ensure that participants were fully familiar with the experiment, they were given 10 practice trials before the formal experiment. The voice stimuli for these practice trials were randomly selected from the 321 voice samples that were not used in the formal trials. The formal experiment consisted of 320 trials (each voice sample was used four times) and was divided into four blocks, each containing 80 trials. The flowchart of a single trial is shown in Figure 1C. First, a black cross appears in the center of the screen for 1,000 ms to remind the participant to focus. Then, a 400 ms audio clip of the “game partner’s” voice (high or low attractiveness) is played. Afterward, the payoff matrix of the two-person Stag Hunt Game designed for this experiment is shown, where the numbers represent the amount of money (in yuan) the participant can earn based on both players’ choices. At this point, the participant must make a choice between “cooperate” or “defect” using the keyboard, pressing the F key for “cooperate” and the J key for “defect.” The participant’s choice will be displayed in red. After a random blank screen of 800–1,000 ms, the result of both players’ choices is displayed. The payoff matrix will show only the number representing the participant’s earnings, while other numbers will be hidden. Participants were informed that the researchers had previously recruited a large number of participants for a behavioral experiment involving the game, collecting their voice data and choice strategies to be used as data for game partners in the EEG experiment. During the EEG experiment, each round of the game randomly presented data from one game partner (voice and choice strategy). In reality, after the participant makes their choice, the computer randomly chooses “cooperate” or “defect” with a 50% probability in each trial. The program sends a marker signal to the EEG data both when the participant is exposed to the voice stimulus (during voice presentation) and when they receive the feedback stimulus (during the game outcome presentation). After the experiment, participants will receive compensation, which consists of a base fee of 65 yuan plus the average earnings from each trial.

### 2.4 EEG recordings

In this experiment, EEG data was collected using a Brain Products EEG amplifier from Germany and a 64-channel Ag/AgCl electrode cap based on the international 10–20 system. The sampling rate was set to 1,000 Hz, with a band-pass filter of 0–100 Hz. The ground electrode was located at AFz on the forehead, and the FCz electrode was used as the reference. Conductive paste was used to connect all electrodes to the scalp. During EEG data recording, impedance between the electrodes and the scalp was kept below 5 kΩ, and behavioral data was recorded simultaneously.

### 2.5 Data analysis

#### 2.5.1 Behavioral data analysis

For behavioral data, the number of times each participant chose “cooperate” under both high-attractiveness and low-attractiveness voice conditions was recorded, and the cooperation rate was calculated. A paired sample *t*-test was used to compare the cooperation rates under high-attractiveness and low-attractiveness voice conditions to determine if there were significant differences.

#### 2.5.2 ERP analysis

The EEG data of each participant was preprocessed using the EEGLAB toolbox (version v2021.0) in MATLAB. First, the reference electrode was converted to the average of the bilateral mastoid electrodes TP9 and TP10. The data was then band-pass filtered between 0.1 and 30 Hz, and a notch filter was used to remove 50 Hz power line interference. The EEG data was segmented based on the markers, from 200 ms before stimulus onset (voice stimulus and outcome feedback stimulus) to 1,000 ms after stimulus presentation. The 200 ms before stimulus onset served as the baseline. The EEG data was visually inspected, and epochs with obvious artifacts were removed. ICA (Independent Component Analysis) was then used to correct artifacts caused by participant movements such as head movements and eye blinks. Epochs with uncorrectable artifacts were manually removed.

After preprocessing the EEG data, the EEG epochs for each stimulus condition were averaged. During the voice stimulus presentation phase, EEG epochs under both high-attractiveness and low-attractiveness voice conditions were averaged separately. During the outcome feedback phase, based on the designed payoff matrix, when the choice outcomes of the participant and the computer counterpart are “cooperate-cooperate” and “defect–defect,” any unilateral change in the participant’s choice would lead to a reduction in participant’s payoff. Thus, these two feedback outcomes are considered “gain.” When the choice outcomes of the participant and the computer counterpart are “cooperate-defect” and “defect-cooperate,” any unilateral change in the participant’s choice would increase participant’s payoff. Thus, these two feedback outcomes are considered “loss.” Thus, there were four feedback conditions: “high attractiveness-gain,” “high attractiveness-loss,” “low attractiveness-gain,” and “low attractiveness-loss,” and the EEG epochs for each of these four conditions were averaged separately.

To explore the brain’s processing of voice attractiveness, during the voice stimulus presentation phase, we conducted statistical analyses on the P2, P3, and LPC components of the EEG. Based on visual inspection of the averaged EEG waveforms and peak latencies, as well as scalp potential distribution, we selected 9 electrodes in the fronto-central region (F1, Fz, F2, FC1, FCz, FC2, C1, Cz, and C2) to analyze the mean amplitudes of the P2 components within the 240–300 ms time window after the voice stimulus presentation. Six electrodes in the centro-parietal region (CP1, CPz, CP2, P1, Pz, and P2) were selected to analyze the mean amplitude of the P3 and LPC component within the selected time windows. The time window for the P3 component was set to 360–450 ms after the voice stimulus presentation, and for the LPC component, it was set to 450–650 ms after the voice stimulus presentation. According to Luck and Gaspelin (2017), averaging electrode sites during analysis can enhance statistical effects. Therefore, we averaged the selected electrode sites for the P2, P3, and LPC components for analysis. Paired-sample t-tests were conducted on the mean amplitudes of the P2, P3, and LPC components elicited under high-attractiveness and low-attractiveness voice conditions.

During the outcome feedback phase, we analyzed the FRN and P300 components. Based on visual inspection of the averaged EEG waveforms and peak latencies, the mean amplitude in the 240–300 ms time window after outcome feedback presentation was used as the index for FRN analysis. For the P300 component, the mean amplitude in the 350–450 ms time window after outcome feedback presentation was used as the analysis index. Based on the scalp potential distribution, 9 electrodes in the fronto-central region (F1, Fz, F2, FC1, FCz, FC2, C1, Cz, and C2) were selected to analyze the FRN component, while 6 electrodes in the centro-parietal region (CP1, CPz, CP2, P1, Pz, and P2) were selected for P300 component analysis. The average values of the selected electrode sites for the FRN and P300 components were analyzed using a 2 (attractiveness: high and low) × 2 (outcome valence: gain and loss) repeated-measures ANOVA. The Greenhouse–Geisser method was used to correct for violations of sphericity (Greenhouse and Geisser, 1959). Multiple comparisons were corrected using the Bonferroni method (Dickhaus, 2014).

#### 2.5.3 ERO analysis

The raw EEG data of each participant was reprocessed using the EEGLAB toolbox (version v2021.0) in MATLAB. First, the reference electrode was converted to the average of the bilateral mastoid electrodes TP9 and TP10. The data was then band-pass filtered between 0.1 and 30 Hz, and a notch filter was used to remove 50 Hz power line interference. The EEG data was segmented based on the markers, from 1,000 ms before stimulus onset (voice stimulus and outcome feedback stimulus) to 2000 ms after stimulus presentation, with the 1,000 ms before stimulus onset serving as the baseline. The EEG data was visually inspected, and epochs with obvious artifacts were removed. ICA (Independent Component Analysis) was then used to correct artifacts caused by participant movements such as head movements and eye blinks. Segments with uncorrectable artifacts were manually removed.

**Figure 2:**
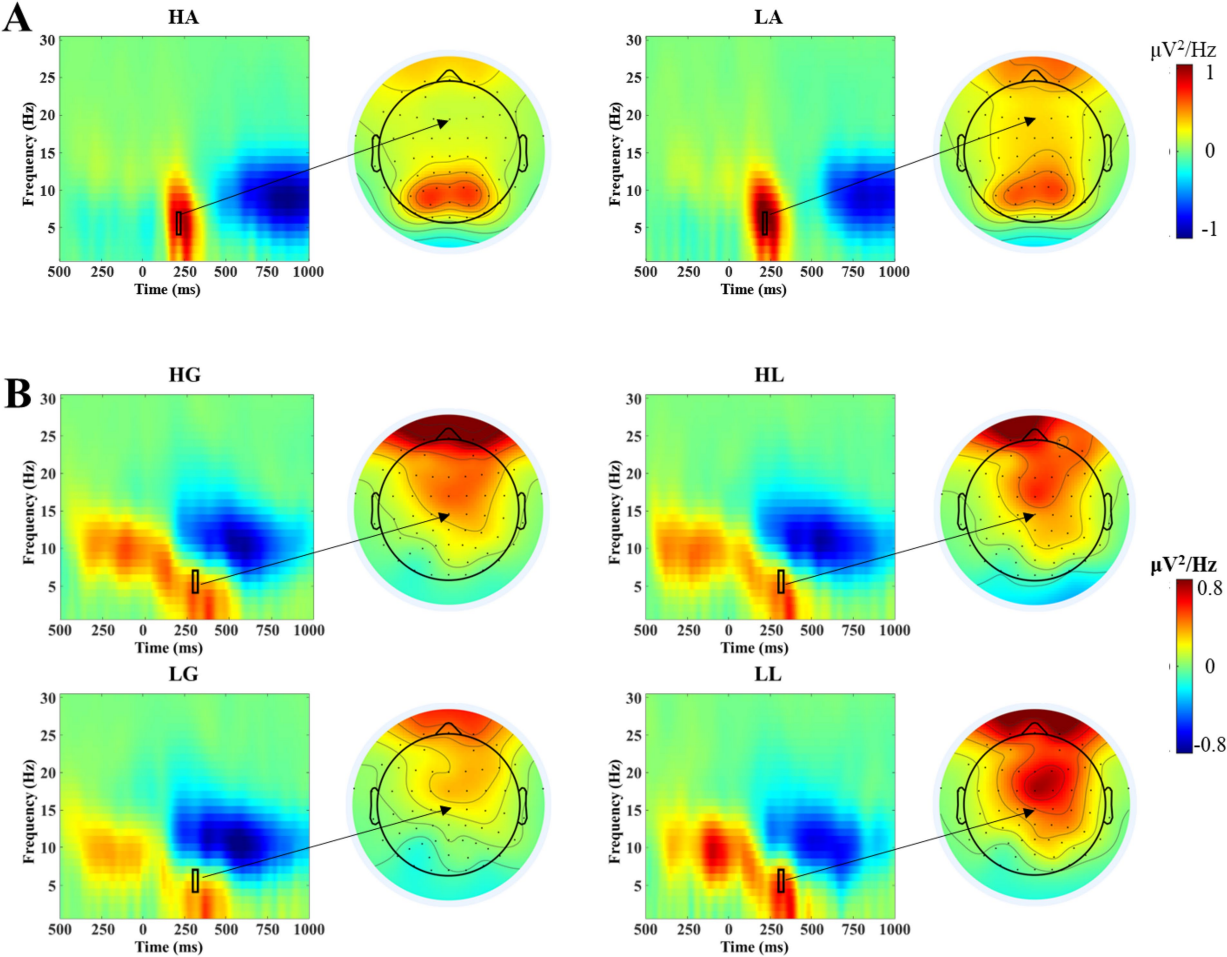
ERO analysis results. **(A)** Time-frequency power plots at the Fz electrode during the voice stimulus presentation phase under high-attractiveness voice (HA) and low-attractiveness voice (LA) conditions. The black rectangle indicates the time window (200–220 ms) and frequency range (4–7 Hz) selected for statistical analysis, with the arrow pointing to the corresponding scalp topography. **(B)** Time-frequency power plots at the Cz electrode during the outcome feedback phase under four conditions: high-attractiveness voice-gain (HG), high-attractiveness voice-loss (HL), low-attractiveness voice-gain (LG), and low-attractiveness voice-loss (LL). The black rectangle indicates the time window (280–310 ms) and frequency range (4–7 Hz) selected for statistical analysis, with the arrow pointing to the corresponding scalp topography.

After EEG data preprocessing, the event-related spectral perturbation (ERSP) analysis was performed on the EEG data using Short-Time Fourier Transform (STFT) technology, obtaining an instantaneous energy estimate within the 1–30 Hz frequency range for each time point. This technique first analyzes the EEG data on a single-trial basis, then averages across multiple trials, ultimately yielding oscillatory power values for each condition. These power values are corrected using a baseline of 800–200 ms before the stimulus. We carefully examined the ERSP spectrogram and scalp topography (see Figure 2A) and selected the Fz electrode for analysis. The Fz electrode is located at the midline of the forehead and is commonly used for studying cognitive and emotional processing. It provides an ideal recording position when analyzing the neural processing of voice attractiveness. Based on visual inspection of the ERSP spectrogram and scalp topography, during the voice stimulus presentation phase, the electrode analyzed was Fz. Paired-sample t-tests were conducted on the mean oscillatory power values in the theta band (4–7 Hz) within the 200–220 ms time window after stimulus onset for high-attractiveness and low-attractiveness voice conditions. During the outcome feedback phase, the Cz electrode was analyzed. The average oscillatory energy values in the theta band (4–7 Hz) within 280–310 ms after feedback stimulus presentation were subjected to a 2 (attractiveness: high and low) × 2 (feedback: gain and loss) two-way repeated measures ANOVA. The Greenhouse–Geisser method was used to correct for violations of sphericity (Greenhouse and Geisser, 1959). Multiple comparisons were corrected using the Bonferroni method (Dickhaus, 2014).

## 3 Results

### 3.1 Behavioral results

The results of the paired-sample *t*-test on participants’ cooperation rates under different voice attractiveness conditions showed that the cooperation rate in the high-attractiveness voice condition (*Mean* = 0.519, *SD* = 0.129) was significantly higher than in the low-attractiveness voice condition (*Mean* = 0.481, *SD* = 0.146), *t* (33) = 3.012, *p* = 0.005.

### 3.2 ERP results

#### 3.2.1 Voice stimulus presentation phase

**Figure 3:**
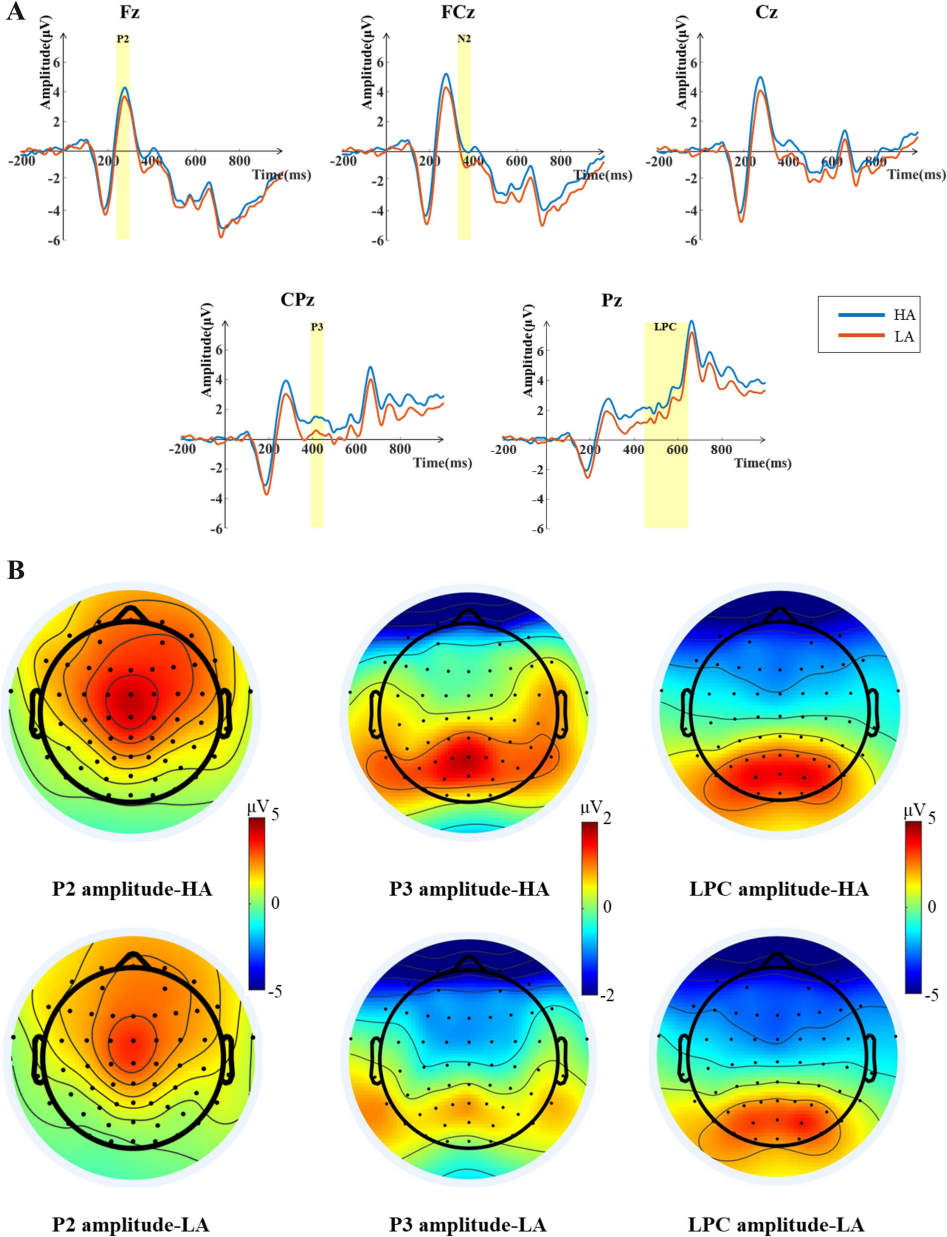
ERP analysis results during the voice stimulus presentation phase. **(A)** Grand averaged ERP waveforms for high-attractiveness voice (HA) and low-attractiveness voice (LA) at the Fz, FCz, Cz, CPz, and Pz electrodes. The shaded areas indicate the time windows selected for the ERP components. The P2 component time window is 240–300 ms, the P3 component time window is 360–450 ms, and the LPC component time window is 450–650 ms. **(B)** Topographic maps of the average amplitudes for the P2, P3, and LPC components induced by high-attractiveness and low-attractiveness voices within the selected time windows.

The results of the paired-samples t-test for the P2 component (see Figure 3) showed the P2 amplitudes elicited under the high-attractiveness voice condition (*Mean* = 3.861 μV, *SD* = 0.567 μV) being significantly larger than that under the low-attractiveness voice condition (*Mean* = 3.000 μV, *SD* = 0.611 μV), *t*(33) = 6.277, *p* = 0.007, *Cohen’s d* = 1.737. The results of the paired-samples t-test for the P3 component showed the P3 amplitudes elicited under the high-attractiveness voice condition (*Mean* = 1.438 μV, *SD* = 0.443 μV) being significantly higher than that under the low-attractiveness voice condition (*Mean* = 0.490 μV, *SD* = 0.419 μV), *t*(33) = 3.193, *p* = 0.003, *Cohen’s d* = 1.731. The results of the paired-samples t-test for the LPC component indicate that the LPC component induced under the high-attractiveness voice condition (*Mean* = 2.134 μV, *SD* = 0.617 μV) was significantly higher than that induced under the low-attractiveness voice condition (*Mean* = 1.354 μV, *SD* = 0.586 μV), *t*(33) = 2.531, *p* = 0.016, *Cohen’s d* = 1.798.

#### 3.2.2 Outcome feedback phase

**Figure 4:**
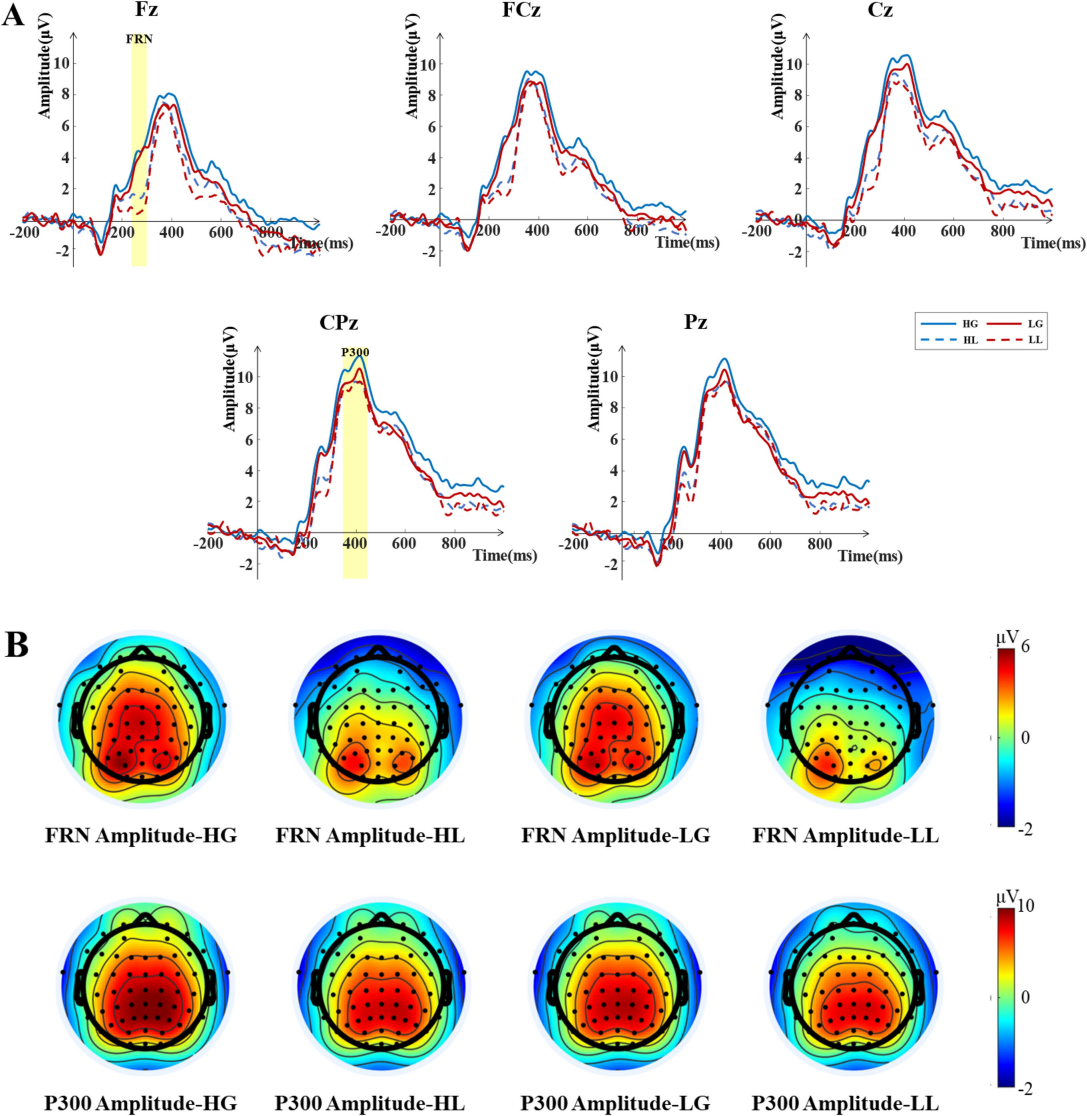
ERP analysis results during the outcome feedback phase. **(A)** Grand averaged ERP waveforms for the four outcome conditions: high-attractiveness voice—gain (HG), high-attractiveness voice—loss (HL), low-attractiveness voice—gain (LG), low-attractiveness voice—loss (LL) at the Fz, FCz, Cz, CPz, and Pz electrodes. The shaded areas indicate the time windows selected for the ERP components. The time window for the FRN component is 240–300 ms, and for the P300 component, it is 350–450 ms. **(B)** Topographic maps of the average amplitudes for the FRN and P300 components elicited by the four outcome conditions within the selected time windows.

The results of the two-way repeated measures ANOVA for the FRN amplitudes (see Figure 4) showed a significant main effect of attractiveness [*F*(1, 33) = 4.438, *p* = 0.043, *η_p_^2^* = 0.119], with the FRN amplitudes elicited under the low-attractiveness voice condition (*Mean* = 3.177 μV, *SD* = 0.894 μV) being significantly more negative than that elicited under the high-attractiveness voice condition (*Mean* = 3.785 μV, *SD* = 0.937 μV). The main effect of outcomes feedback was also significant [*F*(1, 33) = 34.562, *p* < 0.001, *η_p_*^2^ = 0.512], with the FRN amplitudes elicited by loss feedback (*Mean* = 2.122 μV, *SD* = 0.873 μV) being significantly more negative than that elicited by gain feedback (*Mean* = 4.841 μV, *SD* = 0.991 μV). The interaction between attractiveness and feedback was not significant, *F* (1, 33) = 1.361, *p* = 0.252, *η_p_*^2^ = 0.040.

The results of the two-way repeated measures ANOVA for the P300 amplitudes (see Figure 4) showed a marginally significant main effect of attractiveness [*F*(1, 33) = 3.962, *p* = 0.055, *η_p_^2^* = 0.107], with the P300 amplitudes elicited under the high-attractiveness voice condition (*Mean* = 9.947 μV, *SD* = 0.938 μV) being significantly higher than that elicited under the low-attractiveness condition (*Mean* = 9.431 μV, *SD* = 0.902 μV). The main effect of outcomes feedback was also significant [*F*(1, 33) = 4.397, *p* = 0.044, *η_p_*^2^ = 0.118], with the P300 amplitudes elicited by gain feedback (*Mean* = 9.834 μV, *SD* = 0.913 μV) being significantly higher than that elicited by loss feedback (*Mean* = 9.055 μV, *SD* = 0.907 μV). The interaction between attractiveness and feedback was not significant, *F*(1,33) =1.418, *p* = 0.243, *η_p_*^2^ = 0.047.

### 3.3 ERO analysis results

#### 3.3.1 Voice stimulus presentation phase

The results of the paired-samples *t*-test on the theta band power during the voice stimulus presentation phase (see Figure 2A) showed the theta band power produced by low-attractiveness voice (*Mean* = 1.004 μV^2^/Hz, *SD* = 0.190 μV^2^/Hz) being significantly higher than that produced by high-attractiveness voice (Mean = 0.725 μV^2^/Hz, SD = 0.150 μV^2^/Hz), *t*(33) = 2.282, *p* = 0.029, *Cohen’s d* = 0.713.

#### 3.3.2 Outcomes feedback phase

The results of the two-way repeated measures ANOVA on the theta band power during the outcome feedback phase (see Figure 2B) showed that the main effect of attractiveness was not significant [*F*(1, 33) = 0.020, *p* = 0.889], and the main effect of feedback was also not significant [*F*(1, 33) = 2.003, *p* = 0.166], but the interaction between attractiveness and feedback was significant [*F*(1, 33) = 4.434, *p* = 0.043]. Simple effects analysis showed that under the low-attractiveness voice condition, the theta band power produced by loss feedback (*Mean* = 0.469 μV^2^/Hz, *SD* = 0.140 μV^2^/Hz) was significantly higher than that produced by gain feedback (*Mean* = 0.189 μV^2^/Hz, *SD* = 0.135 μV^2^/Hz). Under the high-attractiveness voice condition, there was no significant difference between the theta band power produced by loss feedback (*Mean* = 0.335 μV^2^/Hz, *SD* = 0.133 μV^2^/Hz) and gain feedback (*Mean* = 0.343 μV^2^/Hz, *SD* = 0.120 μV^2^/Hz), *p* = 0.954.

## 4 Discussion

The aim of this study is to investigate whether and how cooperative behavior in the Stag Hunt Game is influenced by the attractiveness of a partner’s voice, and to explore its neural dynamics using neural electrophysiology methods. The study found that the attractiveness of a partner’s voice indeed influenced participants’ behavioral choices, with different voice attractiveness leading to distinct behavioral outcomes and significant differences in scalp potentials and corresponding frequency band activations.

The behavioral data showed that participants exhibited a higher cooperation rate when playing the game with a partner with a high-attractiveness voice, meaning that they were more willing to cooperate with a high-attractiveness voice partner, even when facing the potential risk of defect. In an ultimatum game experiment by Ma et al. (2015), the results showed that male participants were more likely to accept the offer from a female proposer with an attractive face, indicating a “beauty premium” effect of facial attractiveness in decision-making behavior. Our study’s findings are similar, demonstrating that voice attractiveness also exerts a “beauty premium” effect on cooperative behavior in the Stag Hunt Game. When the game partner’s voice is highly attractive, people tend to associate the high-attractiveness voice with more noble personality traits, leading to a more positive behavioral choice tendency, and thus exhibiting more cooperative behavior. In the Stag Hunt Game, choosing to cooperate is a high-risk behavior. However, when faced with an individual with a high-attractiveness voice, participants had more positive expectations (Wilson and Eckel, 2006), leading them to overlook the potential risks of group interaction behavior (Stirrat and Perrett, 2010), resulting in a higher cooperation rate.

During the voice presentation phase, we found that high-attractiveness voices elicited a larger P2 amplitude compared to low-attractiveness voices. The P2 component is believed to be associated with target stimulus classification (Amin et al., 2023) and reflects attention in the 250–350 ms window after stimulus presentation (Antal et al., 2001). In auditory research, it has been shown that the P2 component reflects perceptual-level processing of stimuli by listeners (Paulmann and Kotz, 2008). Compared to low-attractiveness voices, participants may have allocated more attention to high-attractiveness voices at the perceptual level, thus inducing larger P2 amplitudes for high-attractiveness voices.

The P3 component represents the brain’s sustained attention process to a stimulus and is also believed to be related to response decision-making (Luck and Kappenman, 2012; Reed et al., 2022). Research on facial attractiveness has shown that the recognition of facial attractiveness influences the response decision-making process (Ma et al., 2015). In this study, high-attractiveness voices elicited a larger P3 amplitude, indicating participants’ sustained attention to high-attractiveness voices, which in turn guided the subsequent response decision-making process. A recent study by Yuan et al. (2024) also found that high-attractiveness voices induced larger P2 and P3 amplitudes compared to low-attractiveness voices, but they used long vocal stimuli lasting 800–1,200 ms. This study demonstrates that participants can perceive the attractiveness of the voice with a 400 ms short vocal stimulus.

Additionally, between 450 and 650 ms of voice stimulus presentation, high-attractiveness voices induced larger LPC amplitudes than low-attractiveness voices. This is consistent with previous research, where studies on facial attractiveness found that high-attractiveness faces induced larger LPC amplitudes compared to low-attractiveness faces (Ma et al., 2015; Schacht et al., 2008). A recent study by Li et al. (2024) also demonstrated this, suggesting that high-attractiveness faces induce larger LPC amplitudes because they provide higher reward value to the participants. In this study, high-attractiveness voices may also provide participants with a higher perceived reward value. The LPC component is also related to emotional processing (Werheid et al., 2007; Presti et al., 2023), and high-attractiveness voices may evoke more significant emotional stimuli. Research by Schindler and Straube (2020) showed that when attention is focused on emotion-related features, the LPC amplitude increases. In this study, when high-attractiveness voices were presented, participants may have experienced more positive emotions, leading to greater attention, which resulted in larger LPC amplitudes compared to low-attractiveness voices.

The FRN component is often associated with negative events, such as financial losses, negative emotions, and unfair distribution schemes, all of which can elicit a more negative FRN amplitude (Ma et al., 2015; Wu and Zhou, 2009). In a study by Tao et al. (2023), it was found that decision-makers elicited a larger FRN amplitude in negative emotional contexts compared to neutral emotional contexts. The authors suggested that the emotional context influenced the outcome evaluation process in its early stages. In the outcome feedback phase of our experiment, we found that participants elicited a more negative FRN amplitude under the low-attractiveness voice condition, likely because the low-attractiveness voice induced negative emotions in the participants, thereby affecting the outcome evaluation process in its early stages. Previous studies have shown that the FRN component is sensitive to outcome valence, with negative outcomes eliciting a more negative FRN amplitude than positive outcomes when individuals make decisions (Hewig et al., 2007; Peng et al., 2024). Our study produced similar results, with loss feedback eliciting a more negative FRN amplitude compared to gain feedback. Furthermore, the FRN component is thought to be related to reinforcement learning (Bauer et al., 2024; Holroyd and Coles, 2002), with the theory suggesting that when the outcome does not match the expectation, a larger FRN amplitude is elicited. From the participants’ subjective desires, they were more eager to see the outcome of the game with high-attractiveness voice partners and more interested in benefiting from the game. When the outcome violated their expectations, it induced more negative FRN amplitudes. Therefore, the low-attractiveness voice condition and loss feedback elicited a more negative FRN deflection.

The analysis results of the P300 component showed that both the main effects of attractiveness and outcome feedback were significant, with high-attractiveness voices and gain feedback eliciting larger P300 amplitudes. Previous studies have shown that positive outcomes elicit larger P300 amplitudes than negative outcomes (Zhang et al., 2022; Leng and Zhou, 2010; Wu and Zhou, 2009), and our study confirmed this finding. The P300 component typically represents motivational levels, emotional significance, and attention allocation (Leng and Zhou, 2010; Nieuwenhuis et al., 2005; Yeung and Sanfey, 2004). In this study, high-attractiveness voices elicited larger P300 amplitudes, possibly because participants allocated more attention to the game outcomes of partners with high-attractiveness voices compared to those with low-attractiveness voices. Additionally, gaining in the game attracted more of the participants’ attention compared to losses (Ma et al., 2015). Therefore, gain feedback elicited larger P300 amplitudes compared to loss feedback. Additionally, the analysis of the P300 component revealed no significant interaction between voice attractiveness and outcome valence, indicating that the modulation of P300 amplitude by voice attractiveness and outcome valence is independent.

The ERO analysis results showed that during the voice stimulus presentation phase, low-attractiveness voices generated larger theta rhythm oscillations compared to high-attractiveness voices. The study showed that theta activity in the brain is influenced by voice attractiveness. The theta band reflects cognitive control and attentional processing (Nigbur et al., 2011; Senoussi et al., 2022). Evidence suggests that frontal theta oscillations are associated with conflict detection in the brain and that intense conflicts induce greater theta power (Oehrn et al., 2014; Perez-Osorio et al., 2021). In this study, participants may have had a subjective preference for hearing high-attractiveness voices (Ma et al., 2015), leading to significant cognitive conflict when low-attractiveness voices were presented. Therefore, greater theta power was elicited when participants heard low-attractiveness voices. In the outcome feedback phase, under the low-attractiveness voice condition, consistent with previous research, loss feedback produced larger theta band power compared to gain feedback (Cohen et al., 2007). Studies on the role of brain oscillations in conflict and reward situations suggest that theta band oscillations are associated with error and loss processing (Cavanagh et al., 2009). The brain’s processing of negative feedback induces greater theta activity (Cohen et al., 2007), and thus, loss feedback in this study elicited stronger theta activity. When the feedback is a loss, high-attractiveness voices have a rewarding value (Bestelmeyer et al., 2012; O'Doherty et al., 2003), compensating for participants’ negative emotions. Therefore, we believe that high-attractiveness voices distracted participants’ sensitivity to outcome valence, leading to a lack of difference in theta band power between loss and gain feedback under the high-attractiveness voice condition.

The findings of this study are not only academically significant but also have broad potential for practical applications. Understanding the role of voice attractiveness in cooperative behavior can provide valuable insights across multiple fields, especially in social and work environments that require effective cooperation. Voice attractiveness, as a non-verbal signal, can influence the way people interact, playing a key role in teamwork, workplace interactions, and online collaboration platforms. For example, in the workplace, the voice attractiveness of leaders or team members may influence their influence and coordination abilities within the team. Individuals with higher voice attractiveness may find it easier to gain others’ trust and cooperation during decision-making processes, thereby helping the team achieve goals more efficiently. Moreover, in virtual collaborative environments on online platforms, voice attractiveness may influence the cooperative intentions and quality of interactions between team members, especially in the context of remote work. Furthermore, understanding the impact of voice attractiveness on cooperative behavior can provide scientific evidence for improving team dynamics and enhancing team performance. For example, training team members to enhance the attractiveness of their voices in verbal communication may enhance the team’s cooperative spirit and problem-solving abilities. Additionally, the research findings may also be applied in fields such as marketing, public speaking training, and customer service, to optimize communication strategies and improve work efficiency.

## 5 Conclusion

This study is the first to use neuro electrophysiological methods to deeply explore the impact of voice attractiveness on cooperative behavior in the Stag Hunt Game. In the Stag Hunt Game, there is a “beauty premium” effect of voice attractiveness, with individuals showing a higher cooperation rate when facing partners with high-attractiveness voices. During the voice processing phase, high-attractiveness voices induced larger P2, P3, and LPC amplitudes, as well as smaller theta oscillations compared to low-attractiveness voices. In the outcome feedback phase, high-attractiveness voices elicited smaller FRN amplitudes and larger P300 amplitudes compared to low-attractiveness voices, while loss feedback elicited larger FRN amplitudes and smaller P300 amplitudes compared to gain feedback. Only under the low-attractiveness voice condition did loss feedback generate more theta power than gain feedback. Our work provides behavioral and electrophysiological evidence that voice attractiveness influences both decision-making and outcome evaluation processes in the Stag Hunt Game.

## Data Availability

The raw data supporting the conclusions of this article will be made available by the authors, without undue reservation.
